# Cardiorenal Metabolic Modifiers of In-Hospital Outcomes Among Hospitalizations with Acute Kidney Injury

**DOI:** 10.3390/jcm15062407

**Published:** 2026-03-21

**Authors:** Brent Tai, Chijioke Okonkwo

**Affiliations:** BayCare Health System, 3231 McMullen Booth Rd., Safety Harbor, FL 34695, USA; chijioke.okonkwo@baycare.org

**Keywords:** acute kidney injury, heart failure, diabetes mellitus, cardiorenal metabolic disease, in-hospital mortality, dialysis initiation, mechanical ventilation, risk stratification

## Abstract

**Background**: Acute kidney injury (AKI) is a common and high-risk complication of hospitalization that frequently occurs in patients with chronic cardiometabolic disease. Although heart failure (HF) and diabetes mellitus (DM) are prevalent among hospitalized adults and may differentially modify AKI-associated outcomes, their joint impact on in-hospital risk profiles and cumulative burden remains incompletely characterized. **Methods**: We conducted a retrospective analysis of adult hospitalizations complicated by AKI using a nationally representative inpatient database. Hospitalizations were classified into four cardiorenal metabolic phenotypes: AKI alone, AKI with HF, AKI with DM, and AKI with both HF and DM. Primary outcomes included in-hospital mortality, dialysis initiation, and mechanical ventilation. Survey-weighted multivariable logistic regression models incorporating HF, DM, and their interaction were used to estimate adjusted associations and model-based predicted probabilities. Adjusted risks were visualized across outcomes, and a composite burden metric was constructed to summarize cumulative in-hospital adverse events. **Results**: AKI outcomes varied substantially across cardiorenal metabolic phenotypes. HF was consistently associated with higher adjusted mortality and mechanical ventilation risk, whereas DM alone was associated with lower adjusted mortality. A significant interaction between HF and DM was observed regarding dialysis initiation, with a disproportionately higher adjusted risk when both conditions coexisted. Integrated visualization across outcomes demonstrated distinct risk profiles by phenotype, with the combined HF and DM group exhibiting the highest cumulative burden of adverse in-hospital events. **Conclusions**: Among hospitalizations complicated by AKI, the underlying cardiorenal metabolic status is associated with marked heterogeneity in in-hospital outcomes. HF appears to be a dominant modifier of AKI-associated risk, while DM exerts outcome-specific effects and synergistically increases the risk of dialysis initiation when combined with HF. These findings highlight the importance of incorporating cardiometabolic context into AKI risk stratification approaches and underscore the value of multidimensional in-hospital assessments.

## 1. Introduction

Acute kidney injury (AKI) is a common and clinically consequential complication of hospitalization, affecting a substantial proportion of acutely ill adults and conferring increased risk of short-term morbidity and mortality [1,2]. Even transient episodes of AKI are associated with adverse outcomes, including prolonged hospitalization, the need for organ support, and heightened risk of in-hospital death [3,4]. Importantly, AKI rarely occurs in isolation; rather, it frequently develops in the context of multisystem illness and chronic comorbidity, underscoring its role as a marker of global physiological vulnerability in hospitalized patients.

Heart failure and diabetes mellitus are among the most prevalent chronic conditions encountered in hospitalized adults and are well-established risk factors for the development and progression of AKI [5,6]. Heart failure contributes to renal hypoperfusion, venous congestion, and neurohormonal activation [7], whereas diabetes is associated with microvascular dysfunction and heightened susceptibility to renal injury [8]. These conditions commonly coexist, creating a complex cardiorenal metabolic milieu in the context of which kidney injury may be more severe, less reversible, or more likely to require intensive supportive interventions. Despite their frequent co-occurrence, the combined impact of heart failure and diabetes on AKI-related in-hospital outcomes remains incompletely characterized.

Although prior studies have established that heart failure and diabetes independently increase the risk of AKI and worsen short-term outcomes, several important gaps remain [9,10]. Most investigations have examined these conditions in isolation rather than as overlapping, coexisting exposures, limiting insight into whether their joint presence confers additive or synergistic risk. In addition, much of the existing literature has emphasized relative measures of association, such as odds ratios, which may obscure clinically meaningful differences in absolute risk across patient subgroups. Finally, studies often focus on mortality alone, with less attention to other high-impact outcomes (e.g., dialysis initiation and mechanical ventilation) that reflect the severity of illness and resource-intensive care.

For clinicians managing hospitalized patients with AKI, understanding the absolute risk across multiple outcomes is essential for prognostication and care planning. Adjusted predicted probabilities and absolute risk estimates provide a more intuitive assessment of outcome burden than relative measures alone, particularly when the baseline risk differs substantially across comorbidity profiles. Moreover, the evaluation of outcomes in isolation may lead to underestimation of the overall clinical impact of AKI, as patients frequently experience more than one adverse event during a single hospitalization. In this regard, a multidimensional approach that integrates mortality, dialysis initiation, and mechanical ventilation can be expected to offer a more comprehensive view of in-hospital burden associated with complex cardiorenal metabolic disease.

In this study, we examined the association between cardiorenal metabolic status and in-hospital outcomes among adult hospitalizations complicated by acute kidney injury. Specifically, we evaluated how the presence of heart failure and diabetes mellitus—individually and in combination—was associated with in-hospital mortality, dialysis initiation, and mechanical ventilation. We further assessed whether heart failure and diabetes interact to modify the risk of the considered outcomes, as well as characterizing adjusted absolute risk profiles and cumulative in-hospital burden across cardiorenal metabolic phenotypes. Through this approach, we sought to clarify the heterogeneity in AKI-related outcomes within a nationally representative inpatient population.

## 2. Methods

### 2.1. Study Design and Data Source

This study was a retrospective, cross-sectional analysis of adult hospitalizations using data from the 2022 National Inpatient Sample (NIS), part of the Healthcare Cost and Utilization Project sponsored by the Agency for Healthcare Research and Quality. The NIS is the largest publicly available all-payer inpatient database in the United States which was designed to produce nationally representative estimates of hospitalizations across community hospitals [11]. The database captures discharge-level information from acute care hospitals across multiple regions and incorporates a complex survey design, including stratification, clustering, and discharge-level weights, to enable the generation of national estimates. Each record represents a single hospitalization rather than an individual patient; therefore, repeat admissions for the same individual may be included. As the dataset contains de-identified information, this study was considered exempt from institutional review board oversight by BayCare Health System.

### 2.2. Study Population

The analytic cohort included 1,066,993 hospitalizations among adults aged 18 years or older complicated by acute kidney injury (AKI). AKI was identified using diagnosis codes recorded during the index hospitalization. As laboratory data were unavailable, AKI reflects clinically recognized kidney injury during hospitalization rather than biologically adjudicated AKI based on serial creatinine or urine output criteria; notably, AKI timing, severity by KDIGO stage, and distinction between community- and hospital-acquired AKI could not be ascertained. Hospitalizations involving patients younger than 18 years were excluded. Analyses were conducted at the hospitalization level, recognizing that repeat admissions for the same individual may be included. The final study population was stratified according to the presence or absence of heart failure and diabetes mellitus, forming the basis for all descriptive, unadjusted, and adjusted analyses presented in the tables and figures.

To evaluate the robustness of the findings to potential misclassification and the severity enrichment inherent to administrative data, we conducted pre-specified sensitivity analyses by restricting the cohort to hospitalizations with AKI as the principal diagnosis and to dialysis-requiring AKI hospitalizations, as detailed in the Supplementary Material.

### 2.3. Exposure Definition: Cardiorenal Metabolic Status

The primary exposure of interest was cardiorenal metabolic status, defined by the presence of heart failure (HF) and diabetes mellitus (DM) during the index hospitalization. HF and DM were identified using diagnosis codes recorded in the administrative data. Hospitalizations were classified into four mutually exclusive groups: AKI alone, AKI with HF, AKI with DM, and AKI with both HF and DM. The AKI-alone group served as the reference category for all comparative analyses evaluating differences in baseline characteristics, in-hospital outcomes, and adjusted associations across cardiorenal metabolic phenotypes.

### 2.4. Covariates

Covariates were selected a priori based on clinical relevance and the prior literature and included demographic, socioeconomic, and clinical characteristics. Demographic variables comprised age, sex, and race/ethnicity. Socioeconomic factors included primary payer and ZIP code-level median household income quartile, where the latter reflects the patient’s residential ZIP code relative to the national income distribution (as defined by HCUP). Clinical covariates included the presence of chronic kidney disease, which was included to distinguish the modification of acute risk associated with heart failure and diabetes with respect to baseline renal vulnerability, rather than to estimate the total causal effects of cardiometabolic disease on outcomes. These variables were incorporated into multivariable models to account for differences in baseline risk and to isolate the independent and interactive associations of heart failure and diabetes mellitus with in-hospital outcomes.

As an additional sensitivity analysis, ICD-coded obesity (CMR_OBESE) was incorporated into the adjusted models to assess any potential residual confounding related to adiposity.

### 2.5. Outcomes

The primary in-hospital outcomes of interest were all-cause in-hospital mortality, dialysis initiation, and use of mechanical ventilation. Outcomes were ascertained using diagnosis and procedure codes recorded during the index hospitalization. Dialysis initiation reflected receipt of acute renal replacement therapy during hospitalization, while mechanical ventilation captured invasive ventilatory support. All outcomes were assessed during the same hospitalization and were not mutually exclusive.

### 2.6. Descriptive and Unadjusted Analyses

Baseline demographic, clinical, socioeconomic, and hospital characteristics are summarized across cardiorenal metabolic subgroups using survey-weighted means and proportions. Unweighted discharge counts are also reported to describe the underlying sample size. Balance across groups was assessed using standardized mean differences (SMDs), with values greater than 0.20 indicating meaningful imbalance. Unadjusted absolute risks for each outcome were calculated according to cardiorenal metabolic status, and absolute risk differences were estimated relative to the AKI-alone group.

### 2.7. Multivariable Modeling

Survey-weighted multivariable logistic regression models were constructed to evaluate the independent and interactive associations of heart failure and diabetes mellitus with each in-hospital outcome. Models included main effects for heart failure and diabetes mellitus as well as an interaction term between the two conditions. Separate models were fit for in-hospital mortality, dialysis initiation, and mechanical ventilation, adjust for demographic, socioeconomic, and clinical covariates. Adjusted odds ratios with 95% confidence intervals are reported.

Sensitivity analyses were conducted to evaluate the stability of effect estimates under alternative cohort definitions and covariate specifications. First, all primary models were re-estimated with additional adjustment for ICD-coded obesity. Second, analyses were repeated after restricting the cohort to hospitalizations with AKI as the principal diagnosis, representing clinically central AKI. Third, analyses were conducted among dialysis-requiring AKI hospitalizations, representing a severity-enriched phenotype. These analyses were performed to assess robustness to potential misclassification and the severity enrichment inherent to administrative AKI definitions.

### 2.8. Adjusted Predicted Probabilities

To facilitate interpretation of the absolute risk across cardiorenal metabolic phenotypes, model-based adjusted predicted probabilities were estimated for each outcome. Predicted probabilities and corresponding 95% confidence intervals were derived from survey-weighted multivariable logistic regression models using marginal standardization to the covariate distribution of the overall AKI analytic cohort. Because predicted probabilities were estimated using marginal standardization to the overall cohort rather than within-subgroup covariate distributions, adjusted risk estimates may differ from unadjusted subgroup proportions. These estimates were used to compare the adjusted risks of in-hospital mortality, dialysis initiation, and mechanical ventilation across the four cardiorenal metabolic groups.

### 2.9. Visualization and Composite Outcome Assessment

Adjusted predicted probabilities were visualized using multiple complementary figure formats to highlight differences in outcome risk across cardiorenal metabolic phenotypes. A heatmap was used to summarize adjusted risks across outcomes and phenotypes, with color intensity reflecting the relative risk magnitude. In addition, a composite measure of adverse in-hospital burden was constructed by integrating the adjusted risks of mortality, dialysis initiation, and mechanical ventilation. As these outcomes are not mutually exclusive, the composite represents an index of expected adverse events per 100 hospitalizations rather than the probability of experiencing any single outcome. As such, this composite is intended to serve as a descriptive summary of in-hospital burden rather than a validated clinical endpoint.

### 2.10. Statistical Considerations

Statistical methods were pre-specified and are described in sufficient detail to permit replication, including variable definitions, model structure, interaction terms, and sensitivity analyses. All analyses accounted for the complex survey design of the database, including discharge-level weights, stratification, and clustering, to produce nationally representative estimates. The statistical tests were two-sided, with a *p*-value less than 0.05 considered statistically significant. Results are reported with 95% confidence intervals, where appropriate.

All statistical analyses were conducted using R (R Foundation for Statistical Computing, Vienna, Austria, version 4.3.1) [12]. Survey-weighted analyses were performed using established survey analysis packages to account for the complex sampling design of the database. Data management, model estimation, and generation of adjusted predicted probabilities and figures were conducted within the same statistical environment.

The detailed administrative code definitions used to identify exposures, outcomes, covariates, and sensitivity cohorts are provided in Supplementary Table S4.

## 3. Results

### 3.1. Study Population and Cardiorenal Metabolic Phenotype Distribution

This study included a nationally representative cohort of adult hospitalizations complicated by acute kidney injury (AKI). The final analytic cohort included 1,066,993 AKI hospitalizations registered nationwide. Hospitalizations were classified into four mutually exclusive cardiorenal metabolic phenotypes based on the presence of heart failure (HF) and diabetes mellitus (DM): AKI alone, AKI with HF, AKI with DM, and AKI with both HF and DM. The AKI alone group constituted the largest proportion of hospitalizations, followed by AKI with DM, AKI with HF, and AKI with combined HF and DM. Weighted proportions reflect national estimates, whereas unweighted counts represent the underlying sample size for each group (Table 1).

Table 1 illustrates baseline demographic, clinical, socioeconomic, and hospital characteristics of adult hospitalizations complicated by acute kidney injury (AKI), stratified by the presence of heart failure (HF) and diabetes mellitus (DM). The final analytic cohort included 1,066,993 AKI hospitalizations nationwide. Values are survey-weighted unless otherwise specified. Unweighted discharge counts and weighted percentages reflect the distribution of AKI hospitalizations across cardiorenal metabolic subgroups. Standardized mean differences (SMDs) are reported relative to the AKI-alone group; for categorical variables, the maximum SMD across categories is shown, with values >0.20 indicating meaningful imbalance.

### 3.2. Baseline Characteristics Across Cardiorenal Metabolic Groups

Baseline demographic, clinical, and socioeconomic characteristics varied across the cardiorenal metabolic phenotypes (Table 1). Hospitalizations involving HF, with or without DM, occurred among older patients and were characterized by a higher prevalence of chronic kidney disease compared with AKI alone. The racial and ethnic composition differed across groups, with higher representation of Black and Hispanic patients observed among hospitalizations involving diabetes mellitus. Socioeconomic characteristics also varied by phenotype, including differences in primary payer distribution and neighborhood income quartiles. Several characteristics demonstrated meaningful imbalance across groups, as reflected by standardized mean differences exceeding 0.20.

### 3.3. Unadjusted In-Hospital Outcomes

Unadjusted in-hospital outcomes varied across cardiorenal metabolic phenotypes (Table 2). Compared with hospitalizations with AKI alone, hospitalizations involving HF demonstrated higher absolute risks of in-hospital mortality and dialysis initiation, with modest differences in mechanical ventilation risk compared with AKI alone. Hospitalizations involving DM without HF were associated with lower unadjusted mortality and mechanical ventilation risk but higher risk of dialysis initiation relative to AKI alone. The combined HF and DM phenotype exhibited the highest unadjusted risk of dialysis initiation and similar unadjusted mortality and ventilation risks compared with AKI alone. The absolute risk differences quantify the magnitude and direction of these differences across phenotypes.

Table 2 presents survey-weighted absolute risks and unadjusted risk differences for key in-hospital outcomes among acute kidney injury hospitalizations, stratified by the presence of heart failure and diabetes mellitus. Absolute risks (%) and absolute risk differences (ARDs, percentage points) are shown relative to the AKI-alone group.

### 3.4. Adjusted Associations, Interaction Effects, and Sensitivity Analyses

After multivariable adjustment, heart failure was independently associated with higher odds of all three in-hospital outcomes, whereas diabetes mellitus demonstrated outcome-specific associations (Table 3). A statistically significant interaction between HF and DM was observed for dialysis initiation, indicating a departure from multiplicativity on the odds scale, such that the joint presence of these conditions was associated with higher odds of dialysis initiation than expected under a multiplicative model without interaction. In contrast, HF–DM interaction terms were not statistically significant for in-hospital mortality or mechanical ventilation, suggesting that their combined effects on these outcomes were largely additive rather than synergistic.

Table 3 evaluates the independent and interactive associations of heart failure and diabetes mellitus with in-hospital mortality, dialysis initiation, and mechanical ventilation among hospitalizations complicated by acute kidney injury. Models include main effects for heart failure (HF) and diabetes mellitus (DM), their interaction term, and adjustment for age, sex, race/ethnicity, primary payer, ZIP code–level income quartile, and chronic kidney disease. Reference categories were absence of HF and DM, White race, Medicare insurance, and lowest ZIP income quartile. ORs greater than 1 indicate higher odds of the outcome.

In sensitivity analyses additionally adjusting for ICD-coded obesity, the magnitude, direction, and statistical significance of associations between heart failure, diabetes mellitus, and in-hospital outcomes were materially unchanged (Supplementary Table S1). Heart failure remained independently associated with higher odds of mortality, dialysis initiation, and mechanical ventilation, while diabetes mellitus retained outcome-specific associations, including lower adjusted mortality risk.

When restricting the cohort to hospitalizations with acute kidney injury as the principal diagnosis, heart failure remained independently associated with higher in-hospital mortality and dialysis initiation, while diabetes mellitus retained an inverse association with mortality (Supplementary Table S2). No significant interaction between heart failure and diabetes mellitus was observed in this subgroup, indicating that the primary findings were not driven by secondary or incidental AKI coding.

Among dialysis-requiring AKI hospitalizations, heart failure was associated with higher in-hospital mortality, whereas diabetes mellitus remained inversely associated with mortality (Supplementary Table S3). In this severity-enriched cohort, the heart failure–diabetes interaction was attenuated and modestly inverse, suggesting effect modification at the highest levels of illness severity rather than a uniform synergistic effect.

### 3.5. Adjusted Predicted Probabilities by Cardiorenal Metabolic Status

**Figure 1:**
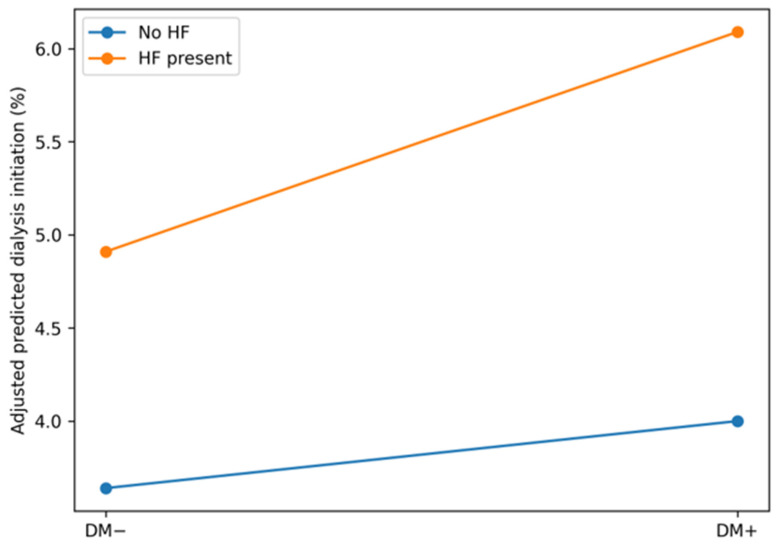
Depicts adjusted predicted probabilities of dialysis initiation among hospitalizations complicated by acute kidney injury (AKI), stratified by diabetes mellitus (DM) status and heart failure (HF). Predicted probabilities were estimated from survey-weighted multivariable logistic regression models including HF, DM, and their interaction, and standardized to the covariate distribution of the AKI analytic cohort. Non-parallel slopes indicate a synergistic association between HF and DM with dialysis initiation.

**Figure 2:**
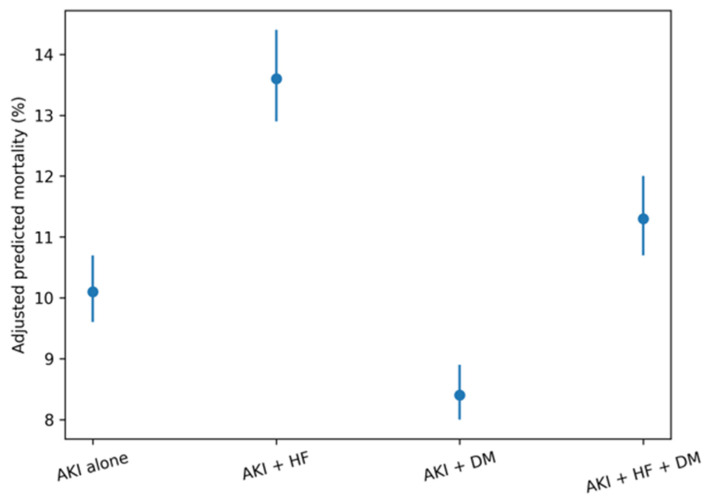
Depicts adjusted predicted probabilities of in-hospital mortality among hospitalizations complicated by acute kidney injury (AKI), stratified by cardiorenal metabolic status. Predicted probabilities and 95% confidence intervals were estimated from survey-weighted multivariable logistic regression models including heart failure (HF), diabetes mellitus (DM), their interaction, and adjustment for demographic, socioeconomic, and clinical covariates.

**Figure 3:**
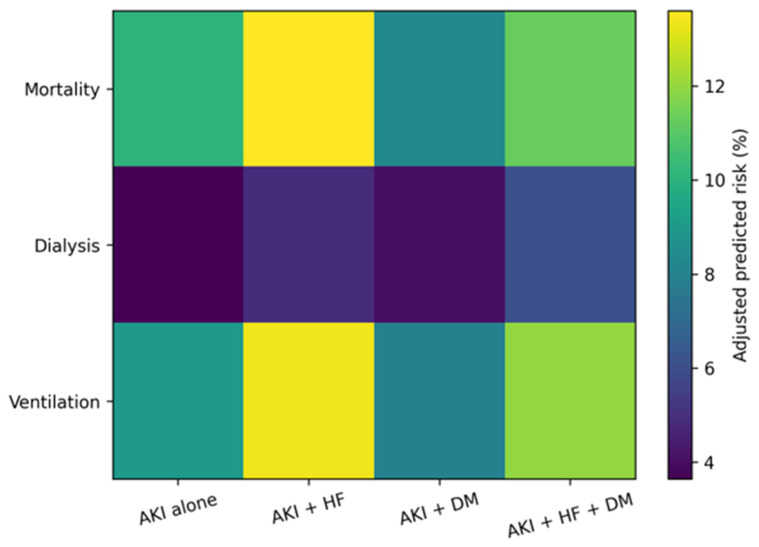
Summarizes adjusted predicted risks of mortality, dialysis, and mechanical ventilation across cardiorenal metabolic subgroups among acute kidney injury hospitalizations. Color intensity reflects the magnitude of adjusted risk. Warmer colors indicate higher adjusted predicted risk. Predicted probabilities were estimated from survey-weighted multivariable logistic regression models including HF, DM, and their interaction, and standardized to the covariate distribution of the AKI analytic cohort.

The model-based adjusted predicted probabilities demonstrated distinct absolute risk profiles across cardiorenal metabolic phenotypes (Table 4; Figure 1). Figure 2 illustrates the absolute adjusted mortality risk to emphasize clinically interpretable risk magnitude, whereas Figure 3 provides a complementary, multidimensional overview of relative risk patterns across mortality, dialysis initiation, and mechanical ventilation. Hospitalizations involving HF were associated with higher adjusted mortality risk and generally higher mechanical ventilation risk compared with AKI alone, whereas those involving DM without HF exhibited lower adjusted mortality risk. The risk of dialysis initiation increased progressively across phenotypes, with the highest adjusted probability observed among hospitalizations with combined HF and DM. Non-parallel risk gradients across HF and DM strata illustrated differential outcome patterns after accounting for demographic, socioeconomic, and clinical factors.

Table 4 presents model-based adjusted predicted probabilities of key in-hospital outcomes across cardiorenal metabolic subgroups to illustrate absolute risk differences after accounting for demographic, socioeconomic, and clinical factors. Predicted probabilities were estimated from survey-weighted multivariable logistic regression models including HF, DM, and their interaction, and standardized to the covariate distribution of the AKI analytic cohort.

Figure 1 Interaction between heart failure and diabetes on adjusted dialysis risk among AKI hospitalizations.

Figure 2 Adjusted predicted in-hospital mortality by cardiorenal metabolic status.

Figure 3 Heatmap of adjusted predicted in-hospital risk across outcomes and cardiorenal metabolic phenotypes.

### 3.6. Integrated Risk Patterns Across Outcomes

When adjusted predicted risks were examined simultaneously across outcomes, distinct cardiorenal metabolic risk patterns emerged (Figure 3). In contrast to the outcome-specific visualizations, Figure 3 provides an integrated overview of relative risk patterns across mortality, dialysis initiation, and mechanical ventilation simultaneously, highlighting multidimensional differences in in-hospital risk profiles according to cardiorenal metabolic status. Hospitalizations involving HF, with or without DM, demonstrated consistently higher adjusted risks across mortality, dialysis initiation, and mechanical ventilation when compared with AKI alone. In contrast, the AKI with DM phenotype showed lower adjusted risk for mortality and mechanical ventilation but intermediate risk of dialysis. The combined HF and DM phenotype exhibited elevated risk across all outcomes, with particularly pronounced risk for dialysis initiation. These patterns highlight the heterogeneity in the outcome profiles across cardiorenal metabolic subgroups after adjustment for measured confounders.

### 3.7. Composite Burden of Adverse In-Hospital Outcomes

**Figure 4:**
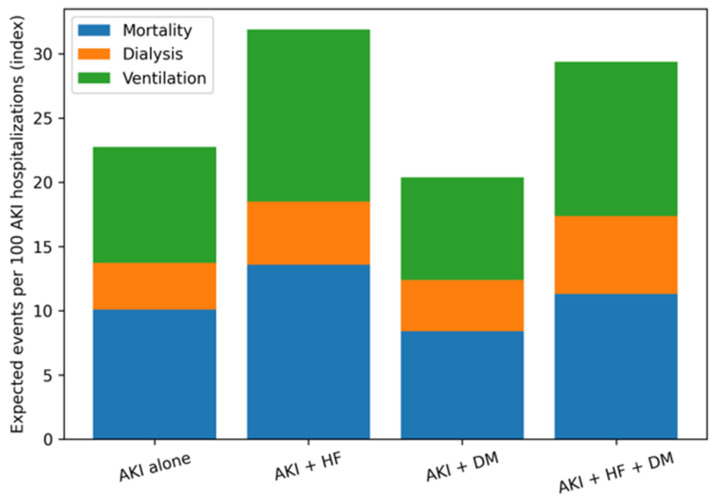
Summarizes the overall burden of adverse in-hospital outcomes across cardiorenal metabolic phenotypes by integrating adjusted risks of mortality, dialysis initiation, and mechanical ventilation. Because outcomes are not mutually exclusive, stacked values represent an index of expected adverse events per 100 hospitalizations rather than the probability of experiencing any single outcome.

The cumulative burden of adverse in-hospital outcomes varied substantially by cardiorenal metabolic status (Figure 4). Hospitalizations with combined HF and DM experienced the highest overall burden, reflecting the aggregation of elevated risks across mortality, dialysis initiation, and mechanical ventilation. Hospitalizations with HF alone also demonstrated a higher composite burden relative to AKI alone, whereas those with DM alone exhibited a lower cumulative burden, which was driven primarily by lower mortality and ventilation risk. As the outcomes were not considered to be mutually exclusive, the composite metric represents the expected number of adverse events per 100 hospitalizations rather than the probability of any single outcome.

Figure 4 Composite burden of adverse in-hospital outcomes by cardiorenal metabolic status.

## 4. Discussion

In this nationally representative analysis of hospitalizations complicated by acute kidney injury, we identified distinct and clinically meaningful risk profiles across cardiorenal metabolic phenotypes. The presence of heart failure was associated with higher adjusted in-hospital mortality, whereas diabetes mellitus alone was associated with lower mortality after adjustment. Our study demonstrates a differential pattern for dialysis initiation, with a synergistic increase in risk when heart failure and diabetes co-occurred. When outcomes were examined collectively, we found that cardiorenal metabolic status was associated not only with the variation in individual outcomes but also with substantial differences in the cumulative burden of adverse in-hospital events. Together, these findings underscore the heterogeneity of AKI outcomes across commonly coexisting cardiometabolic conditions.

The adjusted mortality risk varied substantially according to cardiorenal metabolic status, with heart failure emerging as the dominant determinant of excess risk [13]. Figure 1 shows that hospitalizations involving heart failure—either alone or in combination with diabetes—were associated with higher predicted mortality compared with AKI alone, whereas diabetes without heart failure was associated with lower adjusted mortality risk. This finding should not be interpreted as a protective biological effect of diabetes mellitus; rather, several plausible explanations merit consideration in the context of administrative inpatient data. Hospitalizations involving diabetes may reflect greater baseline healthcare engagement, closer outpatient monitoring, and earlier recognition of renal dysfunction, potentially leading to timelier inpatient management and mitigation of short-term mortality [5,10]. In addition, surveillance and coding practices may preferentially capture AKI at earlier or less physiologically severe stages among patients with diabetes, while more severe AKI in non-diabetic individuals may be under-recognized until later in the hospital course [14,15]. The selection of metabolically stable survivors within the hospitalized AKI population may further contribute to this pattern. Importantly, diabetes was not uniformly associated with favorable outcomes across domains, as it was associated with higher or neutral risk for dialysis initiation and demonstrated context-dependent effects in severity-enriched sensitivity analyses. Together, these considerations underscore that the observed inverse association likely reflects differences in the case distribution, recognition, and care processes rather than a protective effect of diabetes itself.

In contrast to mortality, dialysis initiation demonstrated a clear synergistic association between heart failure and diabetes [16]. Figure 2 shows that the adjusted probability of dialysis initiation increased disproportionately among hospitalizations with both conditions, exceeding the risk observed with either heart failure or diabetes alone. This interaction suggests that the combined presence of cardiac dysfunction and metabolic disease may amplify susceptibility to severe kidney injury and/or lower the clinical threshold for initiating renal replacement therapy. The prominence of this pattern in the dialysis-specific visualization, and its contribution to the overall burden (Figure 4) indicates that the initiation of dialysis represents a particularly sensitive marker of cardiorenal metabolic vulnerability in the setting of AKI.

Patterns of mechanical ventilation further illustrate the differential impact of cardiorenal metabolic conditions on in-hospital severity of illness. Figure 3 shows that hospitalizations involving heart failure demonstrated a higher adjusted risk of ventilation compared with AKI alone, whereas those involving diabetes without heart failure exhibited lower ventilation risk. Unlike dialysis initiation, the coexistence of heart failure and diabetes did not produce a synergistic increase in ventilation risk, suggesting that respiratory failure in this context may reflect additive physiological stress rather than interaction-driven vulnerability. These findings reinforce mechanical ventilation as a marker of global illness severity that, within this observational analysis, was more strongly associated with heart failure than with diabetes mellitus [17].

Examining outcomes in isolation can obscure broader patterns of vulnerability among patients with AKI. Integrating adjusted risks across mortality, dialysis initiation, and mechanical ventilation, Figure 3 highlights distinct cardiorenal metabolic risk profiles that are not apparent when outcomes are considered individually. Hospitalizations involving heart failure, with or without diabetes, clustered toward higher risk across multiple outcomes, whereas diabetes alone was associated with a more selective risk pattern characterized by lower mortality and ventilation risk but intermediate dialysis risk. This visualization underscores the heterogeneity of AKI-associated outcomes and demonstrates the value of multidimensional risk assessment in complex cardiometabolic populations.

The cumulative impact of cardiorenal metabolic status on AKI outcomes is most clearly conveyed by the composite burden shown in Figure 4. Hospitalizations involving both heart failure and diabetes experienced the highest overall burden of adverse in-hospital events, reflecting the aggregation of elevated risks across mortality, dialysis initiation, and mechanical ventilation. In contrast, diabetes alone was associated with a lower cumulative burden—driven primarily by lower mortality and ventilation risk. Summarizing expected adverse events per 100 hospitalizations, the composite metric contextualizes how overlapping cardiometabolic conditions contribute to overall in-hospital burden, beyond what can be inferred from any single outcome.

These findings have important implications for risk stratification and inpatient management of AKI cases in the setting of coexisting cardiometabolic disease. The distinct risk gradients illustrated in our study suggest that heart failure status should be a central consideration when assessing the short-term prognosis among patients with AKI, while diabetes modifies risk in outcome-specific ways. In particular, the disproportionate risk of dialysis initiation observed among patients with combined heart failure and diabetes highlights a subgroup that may benefit from closer kidney monitoring, earlier nephrology involvement, and proactive management of volume and metabolic derangements. More broadly, the composite burden emphasizes that focusing on outcomes in isolation may lead to underestimation of the true clinical impact of overlapping cardiometabolic conditions during AKI hospitalizations.

This study has several notable strengths. The use of a nationally representative inpatient dataset enabled robust estimation of outcome risks across diverse cardiorenal metabolic phenotypes. The analytic approach integrated both relative and absolute risk measures and leveraged adjusted predicted probabilities to enhance clinical interpretability. In addition, the use of multidimensional visualizations—including a heatmap and a composite burden metric—allowed for the synthesis of complex outcome patterns that are difficult to convey through conventional tabular results alone. Together, these features enable a comprehensive and clinically meaningful assessment of AKI risk in the context of common cardiometabolic comorbidities.

This study has several limitations inherent to the use of administrative inpatient data. First, the AKI etiology could not be ascertained, as information on precipitating causes and physiological parameters was unavailable. Accordingly, the findings should not be interpreted as evidence that cardiometabolic conditions such as diabetes mellitus or heart failure cause AKI; rather, these conditions should be viewed as comorbid phenotypes associated with differential risk profiles and in-hospital outcomes among hospitalizations coded for AKI.

Second, the administrative dataset does not distinguish between type 1 and type 2 diabetes mellitus, which may introduce clinical heterogeneity within the diabetes category and could contribute to variation in observed outcome associations.

Third, the identification of AKI relied on administrative diagnosis and procedure codes, which may not fully capture the true incidence or spectrum of AKI in hospitalized patients. Prior studies have demonstrated substantial heterogeneity in AKI recognition and documentation, with a considerable proportion of laboratory-defined AKI episodes not being coded administratively. As a result, administrative definitions may underestimate the occurrence of AKI and preferentially identify more clinically apparent or severe cases, potentially limiting generalizability.

To address these concerns, we conducted multiple severity-enriched sensitivity analyses, including restriction to hospitalizations with AKI as the principal diagnosis and to dialysis-requiring AKI, which yielded findings consistent with those of the primary analysis. Nevertheless, residual confounding related to unmeasured illness severity, AKI timing, or treatment decisions cannot be fully excluded.

In conclusion, among hospitalizations complicated by acute kidney injury, cardiorenal metabolic status is associated with substantial heterogeneity in in-hospital outcomes. Heart failure consistently confers elevated risk across multiple domains, whereas diabetes exerts outcome-specific effects, including a synergistic association with dialysis initiation when coexisting with heart failure. Integrating adjusted risks across outcomes, our study demonstrates how overlapping cardiometabolic conditions shape both individual outcomes and cumulative in-hospital burden. These findings underscore the importance of considering the cardiorenal metabolic context for the prognostic evaluation and management of AKI evaluating prognosis in hospitalized adults.

## Figures and Tables

**Table 1:** Baseline Characteristics of Hospitalizations with Acute Kidney Injury, Stratified by Cardiorenal Metabolic Status.

Variable	AKI Alone	AKI + HF	AKI + DM	AKI + HF + DM	SMD vs. AKI Alone
**Demographics**					
Unweighted count, n (weighted %)	393,909 (36.6%)	184,068 (17.1%)	290,195 (27.0)	207,579 (19.3)	
Age, years (mean ± SE)	64.5 ± 0.14	73.0 ± 0.10	65.8 ± 0.07	71.3 ± 0.06	0.55
Female sex, %	44.2	44.9	44.6	45.0	0.02
Chronic kidney disease, %	30.4	53.7	45.2	69.0	0.84
**Race/ethnicity, %**					
White	68.5	71.9	58.9	63.4	0.20
Black	16.5	17.4	20.3	20.1	
Hispanic	9.3	6.2	13.8	10.5	
Asian/Pacific Islander	2.4	2.0	3.3	2.8	
Native American	0.6	0.4	0.9	0.6	
Other race	2.7	2.2	2.9	2.5	
**Primary payer, %**					
Medicare	56.8	73.1	60.7	73.0	0.35
Medicaid	15.3	10.1	14.8	10.4	
Private insurance	20.4	11.7	17.9	11.9	
Self-pay	4.2	2.3	3.4	1.8	
No charge	0.3	0.2	0.2	0.1	
Other payer	3.0	2.6	3.0	2.8	
**Zip income quartile, %**					
Q1 (lowest)	29.6	29.9	33.5	33.1	0.10
Q2	25.6	25.8	26.2	26.8	
Q3	24.0	24.0	23.1	23.4	
Q4 (highest)	20.8	20.3	17.2	16.7	

**Table 2:** In-hospital outcomes of hospitalizations with acute kidney injury, stratified by cardiorenal metabolic status.

Outcome	AKI Alone (%)	AKI + HF (%)	ARD vs. AKI Alone	AKI + DM (%)	ARD vs. AKI Alone	AKI + HF + DM (%)	ARD vs. AKI Alone
In-hospital mortality	8.63	11.40	+2.77	6.74	−1.89	8.55	−0.08
Dialysis initiation	3.58	4.90	+1.32	4.50	+0.92	7.36	+3.78
Mechanical ventilation	10.20	11.60	+1.40	8.14	−2.06	9.60	−0.60

**Table 3:** Adjusted associations between heart failure, diabetes mellitus, and in-hospital outcomes among hospitalizations with acute kidney injury.

**A. In-Hospital Mortality**			
Variable	Adjusted OR	95% CI	*p*-value
Heart failure (HF)	1.40	1.37–1.43	<0.001
Diabetes mellitus (DM)	0.82	0.80–0.83	<0.001
HF × DM interaction	0.99	0.96–1.02	0.61
**B. Dialysis Initiation**			
Variable	Adjusted OR	95% CI	*p*-value
Heart failure (HF)	1.37	1.32–1.41	<0.001
Diabetes mellitus (DM)	1.10	1.07–1.13	<0.001
HF × DM interaction	1.14	1.10–1.18	<0.001
**C. Mechanical Ventilation**			
Variable	Adjusted OR	95% CI	*p*-value
Heart failure (HF)	1.14	1.11–1.17	<0.001
Diabetes mellitus (DM)	0.88	0.85–0.91	<0.001
HF × DM interaction	0.97	0.93–1.01	0.14

**Table 4:** Adjusted Predicted Probabilities of In-Hospital Outcomes by Cardiorenal Metabolic Status.

Outcome	AKI Alone	AKI + HF	AKI + DM	AKI + HF + DM
In-hospital mortality, %	10.1 (9.6–10.7)	13.6 (12.9–14.4)	8.4 (8.0–8.9)	11.3 (10.7–12.0)
Dialysis initiation, %	3.64 (3.38–3.92)	4.91 (4.56–5.29)	4.00 (3.72–4.29)	6.09 (5.68–6.53)
Mechanical ventilation, %	9.03 (8.64–9.44)	13.4 (12.9–14.0)	8.0 (7.63–8.36)	12.0 (11.5–12.6)

## Data Availability

The data that support the findings of this study are available from the Agency for Healthcare Research and Quality, Department of Health and Human Services of the United States. However, restrictions apply to the availability of these data, which were used under license for the current study, and so are not publicly available. Data are, however, available from the author upon reasonable request and with permission of the Agency for Healthcare Research and Quality.

## Supplementary Materials

The following supporting information can be downloaded at: https://www.mdpi.com/article/10.3390/jcm15062407/s1, Table S1: Obesity-adjusted sensitivity analysis of in-hospital outcomes; Table S2: Sensitivity analysis restricted to hospitalizations with acute kidney injury as the principal diagnosis; Table S3: Sensitivity analysis among dialysis-requiring acute kidney injury hospitalizations; Table S4: Administrative code definitions used to identify exposures, outcomes, and covariates.
