# Supplementary material for: Cardiorenal Metabolic Modifiers of In-Hospital Outcomes Among Hospitalizations with Acute Kidney Injury
Source: J Clin Med. 2026 Mar 21;15(6):2407. doi: 10.3390/jcm15062407 (PMC13027163; doi:10.3390/jcm15062407)
Supplement: Supplementary file 1 [file jcm-15-02407-s001.zip › Supplementary Table S1.pdf]

Supplementary Table S1. Obesity-adjusted sensitivity analysis of in-hospital outcomes

Outcome	Model	Term	Adjusted OR (95% CI)	P value
In-hospital mortality	Primary	Heart failure (HF)	1.39 (1.36–1.42)	<0.001
In-hospital mortality	Primary	Diabetes mellitus (DM)	0.68 (0.66–0.69)	<0.001
In-hospital mortality	Primary	HF × DM interaction	1.09 (1.06–1.12)	<0.001
In-hospital mortality	Sensitivity (+ obesity)	Heart failure (HF)	1.41 (1.38–1.44)	<0.001
In-hospital mortality	Sensitivity (+ obesity)	Diabetes mellitus (DM)	0.69 (0.68–0.71)	<0.001
In-hospital mortality	Sensitivity (+ obesity)	Obesity (ICD-coded)	0.83 (0.82–0.85)	<0.001
In-hospital mortality	Sensitivity (+ obesity)	HF × DM interaction	1.09 (1.06–1.13)	<0.001
Dialysis initiation	Primary	Heart failure (HF)	1.39 (1.35–1.44)	<0.001
Dialysis initiation	Primary	Diabetes mellitus (DM)	1.00 (0.97–1.02)	0.778
Dialysis initiation	Primary	HF × DM interaction	1.20 (1.16–1.25)	<0.001
Dialysis initiation	Sensitivity (+ obesity)	Heart failure (HF)	1.39 (1.34–1.43)	<0.001
Dialysis initiation	Sensitivity (+ obesity)	Diabetes mellitus (DM)	0.99 (0.96–1.02)	0.474
Dialysis initiation	Sensitivity (+ obesity)	Obesity (ICD-coded)	1.06 (1.03–1.08)	<0.001
Dialysis initiation	Sensitivity (+ obesity)	HF × DM interaction	1.20 (1.15–1.25)	<0.001
Mechanical ventilation	Primary	Heart failure (HF)	1.59 (1.55–1.62)	<0.001
Mechanical ventilation	Primary	Diabetes mellitus (DM)	0.76 (0.75–0.78)	<0.001
Mechanical ventilation	Primary	HF × DM interaction	1.08 (1.05–1.11)	<0.001
Mechanical ventilation	Sensitivity (+ obesity)	Heart failure (HF)	1.57 (1.54–1.61)	<0.001
Mechanical ventilation	Sensitivity (+ obesity)	Diabetes mellitus (DM)	0.76 (0.74–0.77)	<0.001
Mechanical ventilation	Sensitivity (+ obesity)	Obesity (ICD-coded)	1.09 (1.07–1.11)	<0.001
Mechanical ventilation	Sensitivity (+ obesity)	HF × DM interaction	1.08 (1.05–1.11)	<0.001

Supplementary Table S1 shows the sensitivity analysis evaluating the association of heart failure, diabetes mellitus, and their interaction with in-hospital outcomes after additional adjustment for ICD-coded obesity among AKI hospitalizations. Models adjusted for age and chronic kidney disease; obesity was added in sensitivity models.
