# Supplementary material for: Cardiorenal Metabolic Modifiers of In-Hospital Outcomes Among Hospitalizations with Acute Kidney Injury
Source: J Clin Med. 2026 Mar 21;15(6):2407. doi: 10.3390/jcm15062407 (PMC13027163; doi:10.3390/jcm15062407)
Supplement: Supplementary file 1 [file jcm-15-02407-s001.zip › Supplementary Table S2.pdf]

Supplementary Table S2. Sensitivity analysis restricted to hospitalizations with acute kidney injury as the principal diagnosis

Outcome	Term	Adjusted OR (95% CI)	P value
In-hospital mortality	Heart failure (HF)	1.98 (1.78–2.21)	<0.001
In-hospital mortality	Diabetes mellitus (DM)	0.78 (0.69–0.87)	<0.001
In-hospital mortality	HF × DM interaction	1.01 (0.86–1.18)	0.934
Dialysis initiation	Heart failure (HF)	1.59 (1.48–1.72)	<0.001
Dialysis initiation	Diabetes mellitus (DM)	1.23 (1.16–1.30)	<0.001
Dialysis initiation	HF × DM interaction	1.03 (0.94–1.13)	0.551

Supplementary Table S2 shows the associations of heart failure and diabetes mellitus with in-hospital outcomes among hospitalizations with acute kidney injury as the principal diagnosis. Models adjusted for age and chronic kidney disease; obesity was added in sensitivity models.
