# Supplementary material for: Cardiorenal Metabolic Modifiers of In-Hospital Outcomes Among Hospitalizations with Acute Kidney Injury
Source: J Clin Med. 2026 Mar 21;15(6):2407. doi: 10.3390/jcm15062407 (PMC13027163; doi:10.3390/jcm15062407)
Supplement: Supplementary file 1 [file jcm-15-02407-s001.zip › Supplementary Table S3.pdf]

Supplementary Table S3. Sensitivity analysis among dialysis-requiring acute kidney injury hospitalizations

Outcome	Term	Adjusted OR (95% CI)	P value
In-hospital mortality	Heart failure (HF)	1.24 (1.16–1.32)	<0.001
In-hospital mortality	Diabetes mellitus (DM)	0.70 (0.66–0.75)	<0.001
In-hospital mortality	HF × DM interaction	0.91 (0.83–0.99)	0.032

Supplementary Table S3 shows the associations of heart failure and diabetes mellitus with in-hospital mortality among dialysis-requiring acute kidney injury hospitalizations. Models adjusted for age and chronic kidney disease; obesity was added in sensitivity models.
