# Supplementary material for: Cardiorenal Metabolic Modifiers of In-Hospital Outcomes Among Hospitalizations with Acute Kidney Injury
Source: J Clin Med. 2026 Mar 21;15(6):2407. doi: 10.3390/jcm15062407 (PMC13027163; doi:10.3390/jcm15062407)
Supplement: Supplementary file 1 [file jcm-15-02407-s001.zip › Supplementary Table S4.pdf]

Supplementary Table S4. Administrative code definitions used to identify exposures, outcomes, and covariates

**Variable**	**Data source**	**Code system**	**Codes / indicators used**	**Operational definition**
Acute kidney injury (AKI)	Diagnosis	ICD-10-CM	N17.*	Presence of any AKI diagnosis code in any diagnosis position during the hospitalization
AKI as principal diagnosis	Diagnosis	ICD-10-CM	N17.* in principal diagnosis field	AKI recorded as the primary reason for hospitalization (used in sensitivity analysis)
Heart failure (HF)	Diagnosis	ICD-10-CM	I50.*, I11.0, I13.0, I13.2	Presence of heart failure diagnosis in any diagnosis position
Diabetes mellitus (DM)	Diagnosis	ICD-10-CM	E10.*, E11.*, E12.*, E13.*, E14.*	Presence of diabetes mellitus diagnosis in any diagnosis position
Chronic kidney disease (CKD)	Diagnosis	ICD-10-CM	N18.*	Presence of chronic kidney disease diagnosis in any diagnosis position
Dialysis initiation	Procedure	ICD-10-PCS	5A1D*	Receipt of acute renal replacement therapy during the hospitalization
Dialysis-requiring AKI	Diagnosis + procedure	ICD-10-CM / ICD-10-PCS	AKI (N17.*) + dialysis (5A1D*)	Hospitalizations with AKI that required acute dialysis (used in severity-enriched sensitivity analysis)
Mechanical ventilation	Procedure	ICD-10-PCS	5A19*	Receipt of invasive mechanical ventilation during hospitalization
Obesity	Comorbidity flag	HCUP CMR	CMR_OBESE	HCUP Elixhauser-based comorbidity indicator for obesity

Variable	Data source	Code system	Codes / indicators used	Operational definition
Age	Demographic	NIS core variable	AGE	Age at admission, in years
Sex	Demographic	NIS core variable	FEMALE	Female sex (binary indicator)
Race/ethnicity	Demographic	NIS core variable	RACE	Self-reported race/ethnicity as recorded in NIS
Primary payer	Socioeconomic	NIS core variable	PAY1	Primary expected payer for the hospitalization
ZIP income quartile	Socioeconomic	NIS core variable	ZIPINC_QRTL	Median household income quartile for patient ZIP code (area-level SES proxy)
In-hospital mortality	Outcome	NIS core variable	DIED	All-cause in-hospital mortality
Hospital identifier	Survey design	NIS core variable	HOSP_NIS	Hospital-level cluster identifier
Discharge weight	Survey design	NIS core variable	DISCWT	Discharge-level weight for national estimates
Stratum	Survey design	NIS core variable	NIS_STRATUM	NIS stratification variable
Composite burden of adverse in-hospital outcomes	Derived analytic construct	N/A	Mortality + dialysis initiation + mechanical ventilation	Sum of adjusted predicted probabilities for mortality, dialysis initiation, and mechanical ventilation per 100 hospitalizations; outcomes are not mutually exclusive
Adjusted predicted probabilities	Model-derived	N/A	Survey-weighted logistic regression with marginal standardization	Model-based absolute risk estimates standardized to the covariate distribution of the overall AKI cohort

Supplementary Table S4 showed that all diagnoses were identified using ICD-10-CM codes and all procedures using ICD-10-PCS codes recorded during the index hospitalization. Unless otherwise specified, diagnosis codes could appear in any diagnosis position. Prefix notation (e.g., N17.*) indicates inclusion of all subordinate codes within that category. Comorbidity indicators were derived using HCUP-provided Clinical Classifications and Elixhauser-based comorbidity flags. Adjusted predicted probabilities were estimated from survey-weighted multivariable logistic regression models using marginal standardization to the covariate distribution of the overall AKI cohort. The composite burden of adverse in-hospital outcomes represents a descriptive summary integrating adjusted risks of in-hospital mortality, dialysis initiation, and mechanical ventilation; because these outcomes are not mutually exclusive, the composite reflects an index of expected adverse events per 100 hospitalizations rather than the probability of experiencing a single outcome.
